# Navigating Romantic Relationships and Sexual Intimacy: Challenges and Opportunities for Adolescents and Young Adults With Cancer ‐ A Literature Review of Qualitative Studies

**DOI:** 10.1002/pon.70285

**Published:** 2025-09-19

**Authors:** Nicole Collaço, Céline Bolliger, Charlotte Cairns, Samantha Sodergren, Olga Husson, Winette T. A. van der Graaf, Maria Rothmund‐Grenier, Milou J. P. Reuvers, Gudrun E. Rohde, Anna L. Hurley‐Wallace, Konstantinos Angoumis, Emily I. Holthuis, Lina H. Lankhorst, Catarina S. Padilla, Simone Hanebaum, Connor J. Tyler, Katherine J. Hunt, Kirsty Way, Richard Wagland, Thomas J. Cartledge, Thom Legendal, Silvie H. M. Janssen, Anne‐Sophie Darlington

**Affiliations:** ^1^ School of Health Sciences University of Southampton Southampton England; ^2^ Faculty of Health Sciences and Medicine University of Lucerne Lucerne Switzerland; ^3^ Department of Medical Oncology Netherlands Cancer Institute Amsterdam the Netherlands; ^4^ Department of Public Health Erasmus MC Cancer Institute Erasmus University Medical Center Rotterdam the Netherlands; ^5^ Department of Surgical Oncology Erasmus MC Cancer Institute Erasmus University Medical Center Rotterdam the Netherlands; ^6^ Department of Medical Oncology Erasmus MC Cancer Institute Erasmus University Medical Center Erasmus the Netherlands; ^7^ Department of Psychiatry, Psychotherapy, Psychosomatics, and Medical Psychology University Clinic of Psychiatry II Innsbruck Medical University Innsbruck Austria; ^8^ Department of Health and Nursing Science Faculty of Health and Sport Sciences University in Agder Kristiansand Norway; ^9^ Department of Clinical Research Sorlandet Hospital Sorlandet Norway; ^10^ Marie Curie Palliative Care Research Department Division of Psychiatry University College London London UK; ^11^ Bioscience, Syngenta Crop Protection Syngenta England; ^12^ Faculty of Medicine Academic Geriatric Medicine University of Southampton Southampton UK

**Keywords:** cancer, intimacy, psychosocial, qualitative, romantic relationships, STRONG AYA, young adult

## Abstract

**Objective:**

This review explores the impact of cancer on adolescents' and young adults' (AYAs) romantic and sexual relationships, examining both challenges and opportunities.

**Methods:**

Qualitative studies on AYAs with cancer and/or their partners were searched based on a sub‐set of studies identified in a previous literature review within the EU Horizon STRONG‐AYA project, and supplemented with an additional search on romantic/sexual relationships. Articles were screened, reviewed in Rayyan and data extracted using a structured framework. Study quality was appraised, and findings were synthesised thematically.

**Results:**

Twenty studies were included. Findings revealed how cancer altered AYAs' sense of self, affecting the navigation of romantic relationships and sexual intimacy across four themes: (1) *Romantic relationship development‐connection, isolation and changing priorities*: Disrupted romantic trajectories and fears of being undesirable hindered relationship formation, though some re‐evaluated priorities, deepening emotional connections, (2) *The disclosure dance: Finding the right time and way*: AYAs struggled with when and how to disclose their diagnosis, with anxiety about burdening partners or altering dynamics. Open and honest communication fostered intimacy, (3) *Body image, sexuality, intimacy, and fertility*: Challenges in these areas impacted self‐worth, but also led to sexual empowerment and adapted intimacy, (4) *Partnerships and social support during cancer*: Unsupportive partners heightened isolation, while supportive partners reinforced self‐worth. Many AYAs experienced growth, resilience and a deeper understanding of themselves/their relationships.

**Conclusion:**

Results may inform age‐appropriate interventions to adequately address the unique challenges AYAs with cancer face in navigating romantic relationships and sexual intimacy.

## 1 Introduction

Adolescents and young adults (AYAs), typically defined as 15 to 39 year‐olds [1], are at a stage of life where they undergo significant physical, cognitive, social and emotional changes [2]. According to Erikson's psychosocial development theory [3], AYAs go through the stages of *Identity* versus *Confusion* and *Intimacy* versus *Isolation* which shape their sense of self and connection to others. A cancer diagnosis during this formative period can significantly disrupt these developmental processes, potentially affecting AYAs current and future relationships, and in turn, their long‐term mental wellbeing and quality of life [4].

Research indicates that compared with AYAs with no lived experience of cancer, AYA cancer survivors experience fewer meaningful romantic relationships and face greater difficulties within those relationships, including lower relationship satisfaction and increased distress [5, 6]. This disparity is a consequence of the impact of cancer and its treatment on a young person's body image and sexual identity [5, 7, 8] in terms of both physical (e.g., decreases in sexual desire and functioning, infertility) and psychosocial changes (e.g., feeling sexually undesirable, fear of rejection by their current or future partner) [4]. These vulnerabilities, while common among all AYAs, are particularly intensified by cancer and its treatment impact [9]. Physical changes such as scarring, hair loss, and infertility can lead to feelings of anxiety, uncertainty about the future and isolation, making intimacy more challenging [10, 11].

The relationship status of AYAs at time of diagnosis can significantly shape their experience of cancer and survivorship. Unpartnered individuals during treatment often encounter greater challenges in their romantic lives and overall wellbeing [12, 13], while supportive relationships can mitigate the emotional and psychological stress of a cancer diagnosis, treatment and survivorship [14, 15]. Partnered individuals also face challenges, including changes in intimacy, shifting caregiver roles, and future‐related anxieties (e.g., family planning) [16, 17, 18]. These stressors, intertwined with the individual's developmental transitions and emerging identity can further intensify feelings of isolation and feel overwhelming [19].

While medical advancements have improved survival rates, the long‐term impact of cancer and treatment on romantic relationships and sexual intimacy remains a significant, yet often overlooked concern, with precedence on survival outcomes. Unlike older adults, AYAs may lack established families, social support and coping mechanisms, making these challenges more distinct.

A previous review [9], which predominantly focused on quantitative studies and a thesis [20] of a qualitative study offer valuable insights into the impact of specific aspects of romantic relationships for young adults with cancer (such as sexual intimacy, fertility, romantic relationships in general), and serve as an important foundation to which our review will build upon. To date, no review has synthesised the qualitative literature to provide a comprehensive understanding of the complexities of how cancer and its treatment collectively impact and shape AYAs' romantic and sexual lives, both for those who are unpartnered, those in established relationships, and additionally from the perspective of solely the partners of AYAs with cancer. By focusing on qualitative studies, this review ensures an in depth and nuanced understanding of the romantic and sexual experiences of AYAs with cancer. The objective of this review is to explore the impact of cancer on AYAs romantic and sexual relationships, examining both the challenges, as well as the opportunities that may arise in these contexts, to ultimately inform appropriate interventions to support AYAs in navigating these aspects of their lives.

## 2 Methods

This review is part of a wider project (STRONG AYA) [21], aimed at improving care for AYAs with cancer, in which a comprehensive literature review was conducted to examine outcomes relevant to this group. Full methodology and results are detailed elsewhere [22]. Herein, we report on the methods applicable to a sub‐synthesis on the impact of cancer on AYA romantic relationships and sexual intimacy. A literature review of qualitative studies was chosen because it allows for a focused exploration of the nuanced and complex experiences in these areas.

### 2.1 Search Strategy

For this literature review, we followed The Enhancing Transparency in Reporting the Synthesis of Qualitative Research (ENTREQ) statement [23]. The search for the overarching literature review formed the basis for this sub‐synthesis, with additional inclusion/exclusion criteria to meet this reviews aims (Box 1). The review protocol is registered with OSF Registries (https://osf.io/q49uk). A search string combined terms related to oncology and adolescents and young adults (Supplementary file 1). A search was conducted of five electronic databases; Embase, MEDLINE ALL, Web of Science Core Collection, Cochrane Central Register of Controlled Trials, and Google Scholar [22]. The overarching literature review search was conducted in October 2022, with no date restrictions. This include backward and forward citation tracking of all included papers. Following receipt of the list of articles from the overarching review, NC conducted an additional targeted Google Scholar search for the years 2024–2025 for this sub‐synthesis, with the final search completed in May 2025. Terms for cancer, qualitative and AYA romantic relationships, including key words and synonyms such as *partner*, *spouse* and *dyadic*, *sexual intimacy* were used to tailor the search for this review (Supplementary file 1).

**BOX 1 Inclusion and exclusion criteria.**

1

**Table:** 

***Primary inclusion criterion (for the overarching literature review)***
Population: AYAs (aged 13–40 years inclusive at time of initial cancer diagnosis) or a subset of this age range (e.g., adolescents OR young adults only). The AYA age range were flexibly applied, because lower and upper age limits for AYAs differ per country or per study Mixed samples were included if age‐stratified outcomes were available for the target population (i.e., AYAs; adolescents; young adults) Studies conducted in other study populations, such as healthcare providers, friends, parents, or carers of AYAs, were included only if the participants provided information on the outcomes of AYAs Study population at study participation: On and/or off treatment, including at diagnosis, during treatment, or following treatment (patients and/or survivors). There was no limit for the time since diagnosis for AYA cancer survivors. Patients on maintenance treatment were included Written in English language Any type of malignant tumor Study designs: Prospective intervention studies, randomized controlled trials (RCTs), observational cohort studies, case–control studies, cross‐sectional studies, qualitative studies, registry studies, mixed‐methods studies (qualitative and quantitative methods) Studies focusing on all types of biological, physical, psychological, or social outcomes
***Exclusion criteria (for the overarching literature review)***
Conference abstracts or posters Articles describing study protocols, case reports/series, reviews/meta‐analyses, expert opinions, theoretical papers, policy documents/guidelines, consensus letters, editorials Articles focusing on outcomes not of interest
***Additional inclusion criteria for the romantic relationships sub‐synthesis***
Qualitative studies only Studies where a central focus or a core theme is on the impact of cancer on romantic relationships and/or sexual intimacy among AYAs Studies focusing on the dyad, i.e. AYA with cancer and partner. Partners are defined as someone in an intimate relationship with the individual and may be of any gender or sexual preference, even if they do not live together. This also includes the term ‘spouse’, or ‘wife/husband’ Studies considering partner‐only interviews, however with a focus on the impact of cancer on the relationship and/or sexual intimacy
***Additional exclusion criteria for romantic relationships sub‐synthesis***
Studies where romantic relationships and/or sexual intimacy are not a central theme but rather a minor or peripheral aspect of the broader AYA cancer experience Studies reporting quantitative results only Studies of young adult survivors of childhood cancer (aligned with overarching review) Studies based only on interviews with caregivers who were not partners/spouses (e.g., the caregiver is a parent or friend)

### 2.2 Screening

Initial screening of identified titles was undertaken by a team of 15 reviewers involved in the overarching literature review. The revised Williamson and Clarke taxonomy [24] was used to categorise outcomes of relevance to AYAs into pre‐specified domains. The framework covered 39 outcome domains across 5 core areas: Epidemiology, Physiological/Clinical, Functioning, Resource use, and Adverse events, with relevant subdomains. The lead reviewer of the overarching literature review (SHMJ) provided all articles categorised under the outcome subdomain ‘social functioning’ and one article from ‘role functioning’. These domains were selected because they encompassed articles aligned with the review's aim.

NC and CB screened this list of papers under these domains and selected eligible articles based on the additional inclusion criteria (Box 1). Disagreement of papers for inclusion was resolved through discussion with CC.

#### 2.2.1 Data Extraction and Management

A data extraction document was used to record publication details, study design, as well as demographic and treatment information. This review extracted data on *challenges* and *opportunities* (Box 2 for definitions), to capture a nuanced and balanced understanding of how cancer shapes AYAs' romantic and sexual relationships.

**BOX 2 Definitions of ‘challenges’ and ‘opportunities’ for data extraction.**

1

**Table:** 

**Challenges** *Challenges* refer to the difficulties or obstacles faced by AYAs (whether partnered or unpartnered) and partners (in partner only articles) within romantic relationships and/or sexual intimacy, due to cancer. These challenges may encompass any aspect of the development or maintenance of relationships and/or sexual intimacy that may be negatively affected by the cancer diagnosis and/or treatment impact, such as changes in relationship dynamics, emotional or physical barriers to intimacy, difficulties in communication, difficulties in dating, or any external stress that impact the development or maintenance of relationships. Challenges may also include issues related to managing the impact of cancer on the future of the relationship.
**Opportunities** *Opportunities* refer to the positive adaptations, changes or growth that occur within romantic relationships and/or sexual intimacy, either in existing relationships, from the perspectives of partners or for those who are unpartnered, as a result of the cancer diagnosis. These can include personal growth, new perspectives on relationships, or positive changes in how one approaches dating, intimacy and connection. For those already in relationships, this can include any information related to positive communication changes, stronger sense of self and resilience in dating and/or strengthened connections.

Extraction of the study data was carried out by NC, CB and CC. Reviewer NC double extracted 10 of the included studies in the sub‐synthesis, covering studies from both CB and CC, to improve rigor. Any discrepancies in data extraction were discussed and resolved between NC, CB and CC.

### 2.3 Quality Appraisal

A quality assessment of the studies included was conducted using the Critical Appraisal Skills Program (CASP) for Qualitative Studies checklist [25]. According to Butler et al., [26], the scoring system for the answers to each question was Yes = 1, Can't tell = 0.5, and No = 0. High quality papers were those scoring between 9–10 points, moderate quality papers 7.5–8, and low quality papers less than 7.5. The CASP checklist assessed study design, data collection, analysis, and result reliability. NC and CC appraised the studies independently, with any discrepancies resolved in discussion with CB (Table 1).

**TABLE 1:** Study descriptions and quality assessment of included studies.

Title & authors	Country	Total Sample *N*	Sub sample characteristics (Mean age, [M], range [R], type of cancer, time since diagnosis/treatment‐ if stated)	Additional characteristics (relationship status, mean length of relationship [Mr] in years, sexual orientation, sex, race, ethnicity‐if stated)	Data collection	Data analysis	Quality appraisal score	Aims
Carpentier, M. Y., Fortenberry, J. D., Ott, M. A., Brames, M. J., & Einhorn, L. H. (2011). Perceptions of masculinity and self‐image in adolescent and young adult testicular cancer survivors: Implications for romantic and sexual relationships. Psycho‐oncology, 20(7), 738–745.	USA	*n* = 21	Age at interview‐18–34 years M‐27.1 years Time since treatment completion‐4–36 months (mean 16.5 months) Testicular cancer survivors	Partnered (*n* = 14; defined as dating, engaged, or married) and had remained in a partnership since treatment. Unpartnered and uninvolved in dating since testicular cancer treatment (*n* = 7) Men (*n* = 21)	Semi structured interviews	Thematic analysis	9.5	To examine adolescent and young adult (AYA) testicular cancer survivors' subjective understanding of the impact of cancer in adolescence and young adulthood, with a particular emphasis on romantic and sexual relationships.
Dobinson, K. A., Hoyt, M. A., Seidler, Z. E., Beaumont, A. L., Hullmann, S. E., & Lawsin, C. R. (2016). A grounded theory investigation into the psychosexual unmet needs of adolescent and young adult cancer survivors. Journal of adolescent and young adult oncology, 5(2), 135–145.	Australia	*n* = 11	Age at diagnosis‐15–40 years M‐27.82 years Age at interview‐21–43 years M‐31.73 years AYA cancer survivors (breast, AML, HL, ovarian, sarcoma, testicular)	Single (*n* = 3) Partner (not living together) (*n* = 3) Married (*n* = 3) De facto (*n* = 2) Years in relationship‐ 0.25–16 years Mr‐ 6.41 years Men (*n* = 5) Female (*n* = 6)	Semi structured interviews	Grounded theory	9	To delineate the pathways through which AYA survivor psychosexual unmet needs manifest, and to demonstrate further the types of psychosexual unmet needs experienced by AYAs.
Hauken, M. A., & Larsen, T. M. (2019). Young adult cancer patients' experiences of private social network support during cancer treatment. Journal of clinical nursing, 28(15–16), 2953–2965.	Norway	*n* = 20	M‐ 31.1 years R: 24–35 years Months since end of treatment‐16 months (1–66 months) Young adult cancer survivors (lymphoma, gynecological)	Married/cohabiting (*n* = 11) Divorced/single (*n* = 9) Male (*n* = 5) Female (*n* = 15)	Semi structured interviews	Thematic analysis	10	To explore young adult cancer patients' experiences of support from their private social network during cancer treatment.
Iannarino, N. T., & Palmer‐Wackerly, A. L. (2022). Fertility preservation decision‐making communication between young adult cancer patients and their romantic partners: An application of the DECIDE typology. Health communication, 37(6), 778–789.	USA	Total sample *n* = 24 *n* = 12 YA cancer survivors and YA romantic partner *n* = 12	Age at diagnosis‐25.9 years R: 19–36 years Partners M‐26.4 years R: 18–37 years Leukemia (*n* = 4), testicular (*n* = 3), breast (*n* = 2), mediastinal (*n* = 1), ovarian/uterine (*n* = 1), head/neck (*n* = 1) Time since completion of treatment‐M‐ 3.2 years Range: 1 week–12.5 years	Wife (*n* = 6) Husband (*n* = 3) boyfriend (*n* = 2) Girlfriend (*n* = 1) Among the 9 currently married dyads, 4 were dating, 3 were engaged, and 2 were married at diagnosis. One biological child (*n* = 3) No children (*n* = 9) YA with cancer White (*n* = 11) Black/African American (*n* = 1) Males‐ (*n* = 7) 58.3% Females‐ (*n* = 5) 41.7% Partner White (*n* = 10) Black/African American (*n* = 1) Hispanic/Latino (*n* = 1) Females‐ (*n* = 7) 58.3% Males‐ (*n* = 5) 41.7%	Individual semi structured interviews	Not clear‐dyadic and thematic analysis, employing constructivist grounded theory principles	9	To explore how YA cancer patients and their romantic partners negotiate unique and complex decisions about fertility with one another, their parents, and other family Members.
Panjwani, A. A., Marín‐Chollom, A. M., Pervil, I. Z., Erblich, J., Rubin, L. R., Schuster, M. W., & Revenson, T. A. (2019). Illness uncertainties tied to developmental tasks among young adult survivors of hematologic cancers. Journal of Adolescent and young adult oncology, 8(2), 149–156.	USA	*n* = 53	Age‐ 20–39 years Time since diagnosis‐less than 2 years (*n* = 27) Between 2 and 5 years (*n* = 21) More than 5 years (*n* = 4) Missing (*n* = 1) Young adult hematologic cancer survivors	Single (never married) (*n* = 32) Married/partnered (*n* = 18) Divorced (*n* = 3) Has children (*n* = 6) Heterosexual (*n* = 50) LGBT (*n* = 3)	Semi structured interviews	Abductive thematic analysis	8.5	To: (1) identify perceptions of uncertainty tied to age‐specific tasks that YAs with HCs experience; and (2) understand how their experiences of uncertainties are shaped by the off‐time nature of illness and developmental stage of young adulthood.
Robertson, E. G., Sansom‐Daly, U. M., Wakefield, C. E., Ellis, S. J., McGill, B. C., Doolan, E. L., & Cohn, R. J. (2016). Sexual and romantic relationships: Experiences of adolescent and young adult cancer survivors. Journal of adolescent and young adult oncology, 5(3), 286–291.	Australia	*n* = 43	Mean age at interview‐22.09 years R: 16.96–26.11 years Mean age at diagnosis of survivors‐19.67 years R: 11–25 years Mean time since treatment completion‐9.69 months R: 1–19 months AYA cancer survivors (mixed cancers)	In a relationship (*n* = 16, 38.1%) Single (*n* = 27, 62.8%) Average relationship length‐19.3 months R: 2–36 months	Semi structured interviews *Mixed methods study‐ only reporting on qualitative)	Qualitative descriptive approach (not stated)	9	To understand better how AYAs in early survivorship perceive the quality of their interpersonal relationships and sexual functioning/satisfaction in reference to their cancer experience.
Stinson, J. N., Jibb, L. A., Greenberg, M., Barrera, M., Luca, S., White, M. E., & Gupta, A. (2015). A qualitative study of the impact of cancer on romantic relationships, sexual relationships, and fertility: Perspectives of Canadian adolescents and parents during and after treatment. Journal of adolescent and young adult oncology, 4(2), 84–90.	Canada	*n* = 20	Age at diagnosis‐ 13.2 years R: 8–16 years Age at interview‐M: 15 years R: 12–17 years Time since diagnosis‐1.8 +–1.5 years Time since last treatment for adolescents in remission R: 0.4–2.4 years Adolescents during and after treatment Mixed cancers	Male (*n* = 9) Female (*n* = 9)	Semi structured interviews	Thematic analysis	9	To gain insight into perspectives related to issues of romantic relationships (adolescent relationships not including sex), sexual relationships (relationships involving sex), and fertility from the vantage point of adolescents and parents during and after treatment.
Yoshida, K., & Matsui, Y. (2022). The impact of cancer on romantic relationships and marriage postdiagnosis among young adult cancer survivors in Japan: A Qualitative study. Journal of Adolescent and young adult oncology, 11(2), 146–155.	Japan	*n* = 24	Age at diagnosis‐M‐ 27.1 years R: 15–38 years Age at interview‐ 24–43 years M‐ 35.5 years AYA cancer survivors	Unmarried (*n* = 24) In partnerships when diagnosed but ended the relationships thereafter (*n* = 7) In partnerships when interviewed (*n* = 18) Japanese (*n* = 24) Heterosexual (*n* = 24) Male (*n* = 8) Female (*n* = 16)	Semi structured interviews	Thematic analysis	9	To explore the impact of cancer on romantic relationships and marriage post‐diagnosis among adolescents and young adults (AYAs) who had been diagnosed with cancer in Japan.
Gorman, J. R., Smith, E., Drizin, J. H., Lyons, K. S., & Harvey, S. M. (2020). Navigating sexual health in cancer survivorship: A Dyadic perspective. Supportive care in cancer, 28, 5429–5439.	USA	*n* = 25 young adult cancer survivors *n* = 25 current male partners	Age at diagnosis‐ M‐ 36 years R: 24–39 years ‐ diagnosis at least 6 months prior to interview. Age at interview‐R: 29–42 years Male partners age at interview‐R: 29–50 years Breast cancer survivors	Married (*n* = 17) Living with partner (*n* = 23) Survivors relationship duration (Mr = 8.9 years for < 35 years old) (Mr = 13.0 years for 35–39 years old) Partners relationship duration (Mr = 10.5 years) Sexual orientation not stated (participants were not required to be heterosexual, but were required to have a male partner) Has children (*n* = 12) Breast cancer survivors: White (*n* = 21) American Indian or Alaska Native (*n* = 2) Asian (*n* = 1) Multi racial (*n* = 1) Hispanic (*n* = 2) Male partners White (*n* = 18) Black or African American (*n* = 1) American Indian or Alaska Native (*n* = 2) Other race (*n* = 2) Hispanic/Latinx (*n* = 3)	Semi‐structured, individual telephone interviews	Thematic inductive analysis Theory of dyadic illness Management	9	To examine how young adult breast cancer survivors (YABCS) and their partners appraised and managed their sexual health and intimate relationships after cancer.
Moules, N. J., Estefan, A., Laing, C. M., Schulte, F., Guilcher, G. M., Field, J. C., & Strother, D. (2017). “A tribe apart”: Sexuality and cancer in adolescence. Journal of Pediatric oncology nursing, 34(4), 295–308.	Canada	*n* = 10	Age at diagnosis‐R: 12–20 years Age at interview‐R: 19–26 years Brain tumors (*n* = 2) Acute lymphoblastic leukemia (*n* = 1) Sarcomas (*n* = 2) Aplastic anemia (*n* = 1) Due to type of data collection and analysis, no other demographic information available on survivors of adolescent cancer.	Female (*n* = 7) Male (*n* = 3) Due to type of data collection and analysis, not other demographic information available.	Hermeneutic interviews	Hermeneutic inquiry	9.5	To explore the complexity of issues of sexuality, sexual development, knowledge, expression, and identity in a population of young adult survivors who experienced cancer as adolescents
Robinson, L., Miedema, B., & Easley, J. (2014). Young adult cancer survivors and the challenges of intimacy. Journal of psychosocial oncology, 32(4), 447–462.	Canada	*n* = 53	Age at diagnosis‐M‐ 29.8 years Age at interview‐M‐ 32.8 years Breast cancer (*n* = 13) Thyroid cancer (*n* = 12) Non‐Hodgkin's lymphoma (*n* = 8) Hodgkin's lymphoma (*n* = 6) Other (*n* = 14)	Committed relationship (*n* = 32) Single (*n* = 19) Divorced (*n* = 2) Male (*n* = 14) Female (*n* = 39)	Open‐ended interviews	Constructivist grounded theory	8.5	To explore intimate relationships after a cancer diagnosis, with young adult cancer survivors.
M Russell, A., Galvin, K. M., Harper, M. M., & Clayman, M. L. (2016). A comparison of heterosexual and LGBTQ cancer survivors' outlooks on relationships, family building, possible infertility, and patient‐doctor fertility risk communication. Journal of cancer survivorship, 10, 935–942.	USA	*n* = 56	Age at diagnosis‐R: 14–42 years Age at interview‐R: 22–46+ years Brain (*n* = 2) Breast (*n* = 7) Cervical (*n* = 1) Colon (*n* = 1) Leukemia (*n* = 3) Lymphoma (*n* = 19) Hodgkin's (*n* = 9) Non‐ Hodgkin's (*n* = 8) Unspecified (*n* = 1) Ovarian (*n* = 5) Sarcoma (*n* = 4) Testicular (*n* = 13) Other (*n* = 1) Adolescent and young adult cancer survivors.	LGBTQ+ (*n* = 22) Heterosexual (*n* = 34) Single at diagnosis (*n* = 26) Single at interview (*n* = 15) Non‐Hispanic White (*n* = 48) Asian (*n* = 1) Hispanic (*n* = 3) Not reported (*n* = 3) Mixed White, Native American (*n* = 1)	Semi‐structured telephone interviews	Content and thematic analysis	9	To understand how LGBTQ survivors are similar to or different from heterosexual survivors with respect to cancer treatments' effects on relationships, plans for parenthood, and fertility preservation decision making.
Bentsen, L., Aagesen, M., Bidstrup, P., Hjerming, M., & Pappot, H. (2024). Sexuality, intimacy, and body image among adolescents and young adults with cancer: a qualitative, explorative study. Supportive care in cancer, 32(4), 219.	Denmark	*n* = 12	Age at interview‐M‐ 25 years R: 22–29 years Time since cancer diagnosis‐1 year‐ over 2 years On treatment (*n* = 4) Post treatment (*n* = 8) Oncological cancer (*n* = 8) Haematological cancer (*n* = 4) Adolescent and young adults with cancer.	Heterosexual (*n* = 10) Bisexual (*n* = 2) In a relationship (*n* = 6) Male (*n* = 5) Female (*n* = 7)	Individual semi‐structured interviews	Thematic analysis	9	(1) To explore Danish adolescents and young adults' (AYAs) thoughts concerning sexual health particularly focusing on sexuality, intimacy, and body image throughout a cancer trajectory, (2) to investigate how AYAs experience healthcare professionals address of‐ and respond to sexual health issues, and (3) to identify AYAs' suggestions on how to support conversation about sexual health.
Wirtz, M. R., Ahmad, Z. N., & Ford, J. S. (2024). “What if I die and no one had ever romantically loved me?”: Sexual well‐being in a sample of YA cancer survivors. Journal of cancer survivorship, 18(1), 186–195.	USA	*n* = 35	Age at interview‐M‐ 31.69 years R: 18–39 years Age at diagnosis‐R: 13–38 Active treatment completed between 2 and 5 years prior to interview Breast cancer (*n* = 9) Lymphomas (*n* = 11) Leukemia (*n* = 4) Colorectal (*n* = 1) CNS (*n* = 2) Kidney (*n* = 2) Other (*n* = 5) Thyroid (*n* = 1) Young adult cancer survivors.	African American/Black (*n* = 1) White (*n* = 30) Native American (*n* = 1) Asian American (*n* = 2) Middle Eastern/Alaska Native (*n* = 1) Not listed (*n* = 1) Hispanic/Latino (*n* = 4) Not Hispanic/Latino (*n* = 31) Heterosexual (*n* = 28) LGBTQ+ (*n* = 5) I don't know (*n* = 1) I Prefer not to answer (*n* = 1) Single (*n* = 12) Partnered (*n* = 2) Married/cohabitating (*n* = 19) Single, divorced (*n* = 2) Male (*n* = 4) Female (*n* = 31)	Semi‐structured interviews	Thematic analysis	9	To elucidate potential themes that YA cancer survivors experience that cross *both* sexual functioning and well‐being.
**PARTNER ONLY PAPERS**								
Yoshida, K., Matsui, Y., & Ando, S. (2025). Partners' perspectives on the impact of cancer on romantic relationships and marriage in Adolescent and young adult cancer survivors. Journal of Adolescent and young adult oncology.	Japan	*n* = 10 partners	Age at time of interview‐ 39.1 years Completed treatment (*n* = 7) Under treatment (*n* = 3) Cancer of spouse/partner: Acute myeloid Leukemia (*n* = 1) Gastric cancer (*n* = 3) Acute lymphocytic leukemia (*n* = 1) Breast cancer (*n* = 2) Cervical cancer (*n* = 1) Testicular cancer (*n* = 1) Sarcoma (*n* = 1)	Male (*n* = 8) Spouses of AYA cancer survivors Female (*n* = 2) in romantic relationships. All participants were in heterosexual relationships. Length of relationship R: 3–23 years Median: 5.5 years	Semi‐structured face to face interviews	Thematic analysis	9	To explore the impact of cancer on romantic relationships and marriage from the perspective of partners of adolescent and young adult (AYA) cancer survivors.
Gao, W., Zhang, Q., Wang, D., li, X., Zhang, L., Xu, M., & han, J. (2024). The role expectations of young women as wives after breast cancer treatment: A qualitative study. International Journal of nursing Sciences.	China	*n* = 26 patients *n* = 6 spouses	Age of YA‐M‐ 35.95 ± 3.36 years Age of spouse‐M‐ 37.67 ± 5.28 years Breast cancer	Mean length of marriage‐13.45 ± 4.83 years R: 3–18 years Female (*n* = 20) Male (*n* = 6)	Semi‐structured face‐to‐face interviews	Colaizzi's phenomenological approach	8	To explore the expectations of the wife role that female cancer patients need to fulfill to return to their families
Moules, N. J., Laing, C. M., Estefan, A., Schulte, F., & Guilcher, G. M. (2018). “Family is who they Say they Are” a: Examining the effects of cancer on the romantic partners of adolescents and young adults. Journal of family nursing, 24(3), 374–404. *Not partner only study as the study included the views of one cancer survivor)	Canada	*n* = 2	Partners of adolescents diagnosed with any form of cancer between: Age‐ 14 and 24 years Due to type of data collection and analysis, not other demographic information available Cancer survivors (plus their romantic partners)	Partnered (*n* = 2 couples) Married (*n* = 1 couple) In a relationship (*n* = 1 couple) Due to type of data collection and analysis, not other demographic information available	Interviews	Hermeneutic analysis/inquiry	9.5	To examine the effects of cancer on romantic partners of adolescents and young adults experiencing, or who have experienced, cancer.
Lewis, P. E., Sheng, M., Rhodes, M. M., Jackson, K. E., & Schover, L. R. (2012). Psychosocial concerns of young African American breast cancer survivors. Journal of psychosocial oncology, 30(2), 168–184.	USA	*n* = 33 cancer survivors	Age at diagnosis‐M‐ 37.39 years At least 1‐year post diagnosis Breast cancer	Living with spouse or partner at diagnosis (39%) Has children (45%) Change in relationship due to cancer (*n* = 26) African American (*n* = 33)	Semi‐structured phone interviews	Descriptive analyses	8	To examine psychosocial concerns in African American breast cancer survivors.
Freidus, R. A. (2017). Experiences of men who commit to romantic relationships with younger breast cancer survivors: A qualitative study. Journal of psychosocial oncology, 35(4), 494–512.	USA	*n* = 13 cancer survivors *n* = 13 partners	Female age at diagnosis‐R: 23–41 years Age at interview‐R: 31–46 years Male age at interview: R: 31–50 years	Domestic part (*n* = 1) Dating (*n* = 5) Engaged (*n* = 2) Married (*n* = 4) Length of relationship M‐1 year R: 7 months ‐ > 10 years Caucasian (*n* = 19) Caucasian/Asian (*n* = 2) Hispanic (*n* = 2) Middle Eastern (*n* = 1) Female (*n* = 13) Male (*n* = 13)	Semi‐structured interviews	Phenomenological analysis	8	To examine the experiences of men who committed to romantic relationships with women under 50, post‐breast cancer diagnosis and treatment.
Davies, J., Hannigan, B., & Kelly, D. (2019). The experience of partners supporting adolescents and young adults with cancer. Journal of advanced nursing, 75(11), 2890–2898.	UK	*n* = 3 AYA cancer patients *n* = 3 partners	Three partners‐(19–20 years old) AYAs (19–20 years old) Acute Lymphoblastic Leukemia (*n* = 1) Hodgkin's Lymphoma (relapse) (*n* = 1) Osteosarcoma (*n* = 1) Time since diagnosis‐ 9–18 months	Relationship length‐ 1–2.5 years Male (*n* = 1) Female (*n* = 1)	Interviews	Thematic analysis	8.5	To investigate choice and control in decision making, the role of partners was significant.

### 2.4 Data and Thematic Synthesis

Data on the *challenges* and *opportunities* were extracted from the text. This extracted text was then studied line by line, annotated, and organised following the three stages of thematic synthesis outlined by Thomas and Harden [27]. Stages one and two involved an iterative process of coding the challenges and opportunities and developing descriptive themes. Microsoft Excel was used to record and manage the data. As we were not approaching the data from a particular theoretical stance, we adopted an integration/aggregation, rather than interpretive approach [28]. The third stage involved going beyond the content of the original studies to generate additional understanding and analytical themes. This involved identifying where themes interconnected and additional insights that differed.

## 3 Results

**FIGURE 1:**
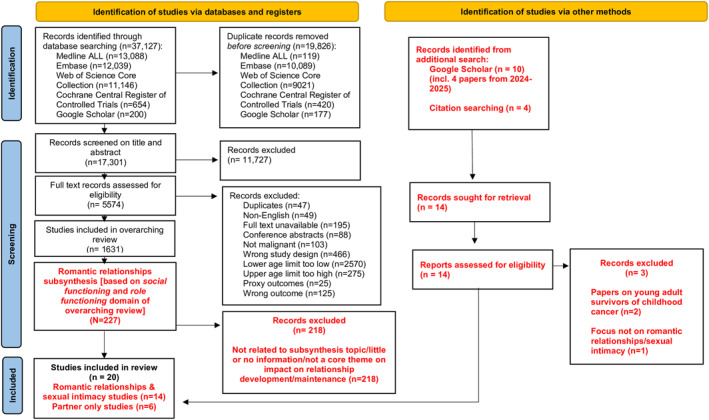
PRISMA flow diagram.

The overarching review included 1631 articles, of which 227 focused on social or role functioning outcomes relevant to this review. After screening, (*n* = 9) articles were deemed eligible for this sub‐synthesis. Further articles (*n* = 12) were included from backward and forward citation tracking, and an additional search. In total, 20 articles met the inclusion criteria for this sub‐synthesis (see Figure 1). Articles included were published between 2011–2025. Across the reviewed articles, (*n* = 14) scored ‘high’ and (*n* = 6) scored ‘moderate’ (Table 1). Two CASP questions frequently elicited “No” responses: “Was the research design justified?” and “Has the researcher critically examined their own role.” The majority of articles were conducted in the USA (*n* = 8), followed by Canada (*n* = 4), Australia (*n* = 2), Japan (*n* = 2), United Kingdom (*n* = 1), Norway (*n* = 1), Denmark (*n* = 1) and China (*n* = 1). Participants were primarily AYA cancer survivors of different cancer types, with interviewee's ages ranging from 15 to 43 years. Partner status varied, with relationship duration ranging from short term partnerships to long term marriages. Ethnicity and sexual orientation were inconsistently reported, though most identified as White and heterosexual. Time since treatment completion varied widely from a few months to over 10 years (Table 1).

This review identified one overarching theme, and four key themes related to how AYAs navigate romantic relationships and sexual intimacy. Central to this is how a cancer diagnosis during adolescence and young adulthood disrupted the typical trajectory of identity formation impacting their sense of self, and subsequently the development and maintenance of romantic relationships and sexual intimacy.

This impact is explored through four key themes and their associated subthemes, highlighting the challenges and opportunities within each (Table 2).

1. Romantic relationship development‐connection, isolation and changing priorities (1 challenge, 1 opportunity)
2. The disclosure dance: finding the right time and way (1 challenge, 1 opportunity)
3. Body image, sexuality, intimacy, and fertility (2 challenges, 3 opportunities)
4. Partnerships and social support during cancer: Balancing caregiving, romantic connection and external pressures (2 challenges, 1 opportunity)

**TABLE 2:** Analytical themes identified in the included studies.

Theme	Insert	Paper
Navigating the development and maintenance of romantic relationships and sexual intimacy with an altered sense of self		
**Romantic relationship development‐ connection, isolation and changing priorities (how cancer shapes identity, impacts on desirability fears, and shifting intimacy priorities)**		
Challenges	Missing out/aging out of key sexual and romantic developmental milestones.	Dobinson et al. 2016 [29]; Wirtz et al. 2024 [30]
	Mismatch between priorities (e.g. survival) and societal expectation for romantic relationships.	Dobinson et al. 2016 [29]; Moules et al. 2017 [7]; Stinson et al. 2015 [8]; Wirtz et al. 2024 [30]
	Increased dependence on family, limiting independence in dating.	Dobinson et al. 2016 [29]
	Unpartnered survivors avoided dating, fearing cancer would affect their desirability. Lost confidence due to fear of being perceived as undesirable or burdensome due to their cancer history, making them hesitant to seek romantic relationships.	Carpentier et al. 2011 [16]; Yoshida et al. 2022 [31]
	The fear of cancer recurrence during checkups created additional stress in relationships, further intensifying emotional strain.	Freidus 2017 [32]; Yoshida et al. 2025 [33]
	Stress of a cancer diagnosis, financial burden, lengthy recovery, and treatment side effects (nausea, fatigue), hospital stays, made it difficult to engage in romantic relationships and sometimes led to breakups, particularly for those in the early stages of a relationship, as these challenges affected intimacy and the ability to form/maintain connections.	Gorman et al. 2020 [34]; Hauken et al. 2019 [35]; Moules et al. 2018 [36]; Robinson et al., 2014 [17]; Stinson et al. 2015 [8]; Yoshida et al. 2022 [31]; Yoshida et al. 2025 [33]
	Rejection or different treatment from potential partners due to cancer.	Lewis et al. 2012 [11]; Yoshida et al. 2022 [31]
	Survivors felt isolated, believing partners could not fully understand their experience.	Carpentier et al. 2011 [16]
Opportunities	Cancer prompted individuals to re‐evaluate their relationships, leading them to prioritise emotional intimacy and view marriage and commitment with greater seriousness, shifting focus away from physical aspects.	Bentsen et al. 2024 [37]; Carpentier et al. 2011 [16]; Freidus 2017 [32]; Moules et al. 2018 [36]; Panjwani et al. 2019 [38]
	Cancer strengthened emotional bonds between partners, fostering a deeper appreciation for their relationship through mutual understanding and shared experiences, with couples often viewing cancer as an obstacle they overcame together.	Carpentier et al. 2011 [16]; Davies et al. 2019 [39]; Freidus 2017 [32]; Gao et al. 2024 [40]; Gorman et al. 2020 [34]; Hauken et al. 2019 [35]; Lewis et al. 2012 [11]; Moules et al. 2018 [36]; Panjwani et al. 2019 [38]; Robertson et al. 2016 [41]; Robinson et al., 2014 [17]
	Sharing vulnerability within a supportive relationship helped AYAs rediscover their self‐worth, attractiveness, and sense of identity.	Davies et al. 2019 [39]; Freidus 2017 [32]
	Cancer prompted participants to reassess their relationship priorities, helping them better understand each other's needs and fostering deeper, more meaningful connections.	Moules et al. 2017 [7]; Moules et al. 2018 [36]; Panjwani et al. 2019 [38]; Robinson et al., 2014 [17]; Yoshida et al. 2022 [31]; Yoshida et al. 2025 [33]
	No change or discernible negative impact on the relationship.	Yoshida et al. 2022 [31]; Yoshida et al. 2025 [33]
**The disclosure dance: Finding the right time and way (navigating conversations about cancer, health history, sexual matters and fertility in relationships)**		
Challenges	Anxiety about when and how to disclose a cancer diagnosis, particularly in early dating/partners anxieties around how to ask the right questions to person with cancer.	Carpentier et al. 2011 [16]; Freidus 2017 [32]; Lewis et al. 2012 [11]; Russel et al. 2016 [42]; Moules et al. 2017 [7]; Panjwani et al. 2019 [38]; Yoshida et al. 2022 [31]
	Disclosing a cancer diagnosis often deterred forming close relationships, as individuals feared rejection or being seen as a burden, particularly in early dating.	Carpentier et al. 2011 [16]; Russel et al. 2016 [42]; Panjwani et al. 2019 [38]; Stinson et al. 2015 [8]; Yoshida et al. 2022 [31]
	Navigating conversations about fertility and family planning was difficult, particularly in uncertain or new relationships.	Yoshida et al. 2022 [31]
	Lack of communication (about sexual matters or emotional issues) led to disconnection/withdrawal/frustration/misunderstandings.	Gorman et al. 2020 [34]; Ianmarino et al. 2022 [43]; Robertson et al. 2016 [41]; Robinson et al., 2014 [17]
Opportunities	Early and upfront disclosure of a cancer diagnosis helped build trust in dating.	Freidus 2017 [32]; Lewis et al. 2012 [11]; Yoshida et al. 2022 [31]; Yoshida et al. 2025 [33]
	Open, honest communication about sensitive topics, along with reciprocal sharing and active listening, fostered trust, emotional closeness, and strengthened relationships.	Carpentier et al. 2011 [16]; Gao et al. 2024 [40]; Ianmarino et al. 2022 [43]; Panjwani et al. 2019 [38]; Robertson et al. 2016 [41]
	Partners adjusted to physical changes by maintaining open communication, allowing them to navigate challenges together.	Freidus et al. 2017 [32]
**Sexuality, intimacy, body image and fertility after cancer (how cancer impacts sexual identity, intimacy, body image and fertility)**		
Challenges	Reduced sexual intimacy made couples feel older than their age.	Dobinson et al. 2016 [29]; Wirtz et al. 2024 [30]
	AYAs felt isolated, missing typical young adult experiences and conversations about sexuality with peers.	Moules et al. 2017 [7]
	Concerns about pleasing partners and the fear of romantic rejection due to sexual inabilities complicated self‐esteem and intimacy.	Bentsen et al. 2024 [37]; Wirtz et al. 2024 [30]
	Survivors perceived threats to their masculinity due to impact on sexual ability.	Bentsen et al. 2024 [37]; Carpentier et al. 2011 [16]; Dobinson et al. 2016 [29]
	Physical changes from cancer symptoms/treatment side effects (erectile dysfunction, vaginal dryness, pain, fatigue reduced libido) complicated maintaining sexual intimacy and could strain committed and casual relationships.	Bentsen et al. 2024 [37]; Dobinson et al. 2016 [29]; Gao et al. 2024 [40]; Gorman et al. 2020 [34]; Lewis et al. 2012 [11]; Robertson et al. 2016 [41]; Robinson et al., 2014 [17]; Stinson et al. 2015 [8]; Wirtz et al. 2024 [30]
	Hurtful reactions about scars from partner affected self‐esteem and intimacy.	Wirtz et al. 2024 [30]
	Concerns about sexual desire and performance contributed to a loss of confidence, leading to anxiety, fear, and vulnerability in relationships.	Robinson et al. 2014 [17]
	Concerns about fertility (uncertainty in future relationships/fearing partners will reject them, feeling guilty not providing biological children) could lead to strained expectations with partners.	Bentsen et al. 2024 [37]; Carpentier et al. 2011 [16]; Dobinson et al. 2016 [29]; Freidus 2017 [32]; Lewis et al. 2012 [11]; Russel et al. 2016 [42]; Panjwani et al. 2019 [38]; Stinson et al. 2015 [8]; Yoshida et al. 2022 [31]; Yoshida et al. 2025 [33]
	Concerns about passing on genetic predispositions created anxiety about family planning.	Freidus 2017 [32]; Yoshida et al. 2022 [31]; Robinson et al. 2014 [17]
	Body image issues (changes like hair loss and scarring, weight fluctuations, stoma) caused individuals to feel self‐conscious, and a loss of self‐confidence, creating barriers for both individuals in relationships and those who were single, making it harder to form or maintain romantic and sexual connections.	Bentsen et al. 2024 [37]; Carpentier et al. 2011 [16]; Freidus 2017 [32]; Gao et al. 2024 [40]; Lewis et al. 2012 [11]; Russel et al. 2016 [42]; Panjwani et al. 2019 [38]; Robertson et al. 2016 [41]; Robinson et al., 2014 [17]; Stinson et al. 2015 [8]; Wirtz et al. 2024 [30]; Yoshida et al. 2022 [31]
Opportunities	Empowerment in sexuality improved intimacy and redefined connections allowing individuals to take control of sexual identity.	Gorman et al. 2020 [34]; Wirtz et al. 2024 [30]
	Filtering out incompatible partners who were unwilling to accept sexual limitation or them as individuals.	Carpentier et al. 2011 [16]; Wirtz et al. 2024 [30]; Yoshida et al. 2022 [31]
	Intimacy played a key role in reclaiming normalcy and fostering self‐acceptance after cancer.	Moules et al. 2017 [7]
	Learning to embrace a new sexual identity, rather than holding onto past frustrations, and valuing inner qualities over physical appearance, fostered self‐acceptance.	Bentsen et al. 2024 [37]; Moules et al. 2017 [7]
	Acceptance from their partner helped rebuild self‐esteem and foster intimacy.	Carpentier et al. 2011 [16]; Freidus 2017 [32]; Lewis et al. 2012 [11]; Robinson et al., 2014 [17]; Wirtz et al. 2024 [30]
	Couples re‐evaluated life goals, particularly around family planning, with some finding comfort in alternatives like adoption.	Russel et al. 2016 [42]
	Redefined intimacy by finding creative ways to connect physically despite changes, such as through kissing, hugging, and using lubricants or estrogen replacement.	Gao et al. 2024 [40]; Gorman et al. 2020 [34]; Lewis et al. 2012 [11]; Robinson et al., 2014
**Partnerships and social support during cancer: Balancing caregiving, romantic connection and external pressures (how cancer shifts relationship roles, and introduced external challenges from family and society)**		
Challenges	AYAs felt isolated and unsupported due to their partner's emotional withdrawal or lack of support.	Dobinson et al. 2016 [29]; Hauken et al. 2019 [35]; Lewis et al. 2012 [11]; Robinson et al., 2014 [17]
	Partners experienced cynicism, anger, and grief, due to impact of cancer which put significant strain on their relationships.	Freidus 2017 [32]; Lewis et al. 2012 [11]
	Some wives felt obligated to maintain traditional roles, such as managing household chores or being the primary decision‐maker, even while recovering from treatment.	Gao et al. 2024 [40]
	Dependence on the partner during treatment placed many responsibilities on them (e.g., physical care, emotional support), which sometimes led survivors to feel like a burden.	Hauken et al. 2019 [35]
	The partner's fear of recurrence and ongoing health concerns sometimes led them to struggle with meeting the survivor's needs, resulting in a lack of support or even abandonment.	Freidus 2017 [32]; Robinson et al., 2014 [17]
	Partners felt pressured to stay in relationships despite challenges, due to societal/family expectations.	Moules et al. 2018 [36]
	Feeling unable to fulfill their roles as partners or meet the expectations of the person with cancer strained relationships, especially in terms of sexual intimacy.	Bentsen et al. 2024 [37]; Gao et al. 2024 [40]; Gorman et al. 2020 [34]
	Partners balanced their own needs with the patient's, sometimes hiding challenges.	Davies et al. 2019 [39]; Freidus 2017 [32]; Gao et al. 2024 [40]; Yoshida et al. 2025 [33]
	Families sometimes opposed survivor's relationships due to concerns about the survivor's wellbeing, the partner might be burdened or cultural expectations around family and filial duties.	Yoshida et al. 2022 [31]
	Partners felt sidelined by family members prioritising the survivor's needs.	Moules et al. 2018 [36]
	Survivor's illness forced partners into new family roles, which sometimes risked the stability of the family unit.	Gao et al. 2024 [40]
Opportunities	Partners provided essential support to AYAs with cancer (practical and emotional support).	Carpentier et al. 2011 [16]; Davies et al. 2019 [39]; Gao et al. 2024 [40]; Hauken et al. 2019 [35]; Robertson et al. 2016 [41]; Robinson et al., 2014 [17]; Stinson et al. 2015 [8]; Wirtz et al. 2024 [30]
	Reassurance and open communication from partners about their needs and the survivor's needs strengthened relationships and minimised conflict.	Davies et al. 2019 [39]; Dobinson et al. 2016 [29]; Gao et al. 2024 [40]; Robertson et al. 2016 [41]; Robinson et al., 2014 [17]
	Partner's support reshaped how individuals viewed themselves, reinforcing their strength and resilience.	Carpentier et al. 2011 [16]; Davies et al. 2019 [39]; Ianmarino et al. 2022 [43]
	Witnessing a partner navigate cancer deepened admiration and mutual respect within relationship.	Davies et al. 2019 [39]; Robertson et al. 2016 [41]; Yoshida et al. 2025 [33]
	Survivors developed a deeper understanding of emotional connection, which fostered greater compassion and intimacy in their relationship.	Moules et al. 2017 [7]

### 3.1 Overarching Theme: Navigating the Development and Maintenance of Romantic Relationships and Sexual Intimacy With an Altered Sense of Self

**FIGURE 2:**
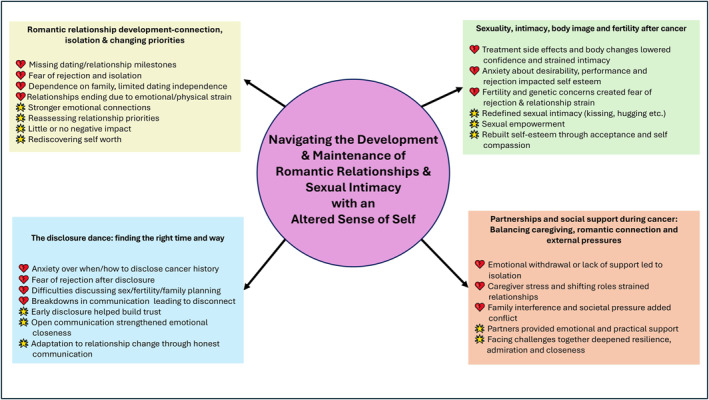
Key themes in review.

Experiencing cancer as an AYA can significantly impact their developing sense of self, which in turn influences their ability to establish and sustain romantic relationships. This often creates a conflict between the desire for physical and emotional connection and the challenges posed by illness and recovery. Figure 2 illustrates this framework, and the four key themes described below elaborate on the different ways this impact is experienced by AYAs.

## 4 Romantic Relationship Development‐Connection, Isolation and Changing Priorities

### 4.1 Challenges

#### 4.1.1 Disrupted Romantic Trajectories

The emotional and physical impact of cancer disrupted the typical developmental trajectory of AYAs [29, 30], often leading to feelings of isolation. Some felt they had reverted to childhood, becoming more dependent on family and disengaging from romantic relationships:

I never really had boyfriends through high school…at the time when you’re supposed to be… creating your own independence… I was spending all of my time with my parents and almost reverted to childhood […] [29].

Conversely, others experienced forced early maturation, prioritising survival over romantic development [7, 8, 29, 30]. This mismatch between their priorities and societal expectations led to a disconnection between their personal needs and societal expectations. AYAs had to balance the necessity of medical treatment with the desire for social engagement, independence and intimacy. This often resulted in an internal conflict along with feelings of loneliness and inadequacy [7, 8, 30]:

And also, I—you know, when I had that feeling of like, oh my God, what if I die and no one had ever romantically loved me? I really did want to be involved with somebody…[30].

Fear of being seen as undesirable or a burden due to their cancer history left many, especially unpartnered AYAs, hesitant to date [16, 31]. Treatment side effects (e.g., fatigue, nausea), hospitalisation, and stigma‐related fears about cancer further contributed to social withdrawal. The stress of diagnosis and lengthy recovery sometimes resulted in breakups, particularly in early‐stage relationships [8, 17, 31, 34, 35, 36].

### 4.2 Opportunities

#### 4.2.1 Re‐Evaluation and Redefined Priorities

Cancer prompted AYAs to re‐evaluate their relationships and self‐perception, accelerating a reassessment of their values and goals [16, 32, 36, 37, 38] often not experienced by their peers until later in life. For some young adults with cancer, this led to prioritisation of emotional intimacy and view of commitment, including marriage with greater seriousness, which brought them closer together [16]. Similarly, Freidus et al. [32] found that some men who were dating breast cancer survivors prioritised love and emotional connection over the diagnosis itself:

You’re not dating someone’s cancer. You’re dating the person [32].

This shift in priorities strengthened emotional bonds between partners, fostering mutual understanding and a deeper appreciation of their relationship. Many couples viewed cancer as an obstacle they overcame together, which deepened their connection [11, 16, 17, 32, 33, 35, 36, 38, 39, 40, 41, 44]:

It (cancer) brought us closer [36].

Additionally, sharing vulnerability within supportive relationships allowed AYAs to rediscover their self‐worth and attractiveness, significantly impacting on their self‐perception and relationship development [32, 39, 44].

While some relationships strengthened after a cancer diagnosis, in some cases, there was no discernible negative impact [31, 33, 44].

## 5 The Disclosure Dance: Finding the Right Time and Way

### 5.1 Challenges

#### 5.1.1 Disclosure Dilemmas

Disclosing a cancer diagnosis was a significant challenge for young adult survivors, and for some, was a source of anxiety [16, 38], deterring them from forming close relationships [31]. This fear manifested in anxieties about being pitied, perceived as weak or undesirable, burdening partners, or being defined by their illness [16, 38]:

[…] I think about it sometimes when I am like dating online, like when do I tell the guy that I have cancer, […], is he gonna like wanna not be with me because maybe he’s afraid? Like he’s gonna have to take care of me in like my old age, if I get to old age […]. [38].

Uncertainty about when, how and to whom they should disclose information about their cancer added complexity to forming new relationships [7, 11, 16, 31, 32, 38, 42], and also extended to casual dating/sexual partners [38]. Age and relationship status also influenced disclosure experiences. Unpartnered survivors expressed anxieties about burdening potential partners with their cancer history, fearing rejection, stemming from the uncertainty of their future health and its potential impact on relationships [8, 16, 31, 38, 42]. Cultural expectations regarding marriage exacerbated concerns about disclosure, particularly the fear of being perceived as *“damaged goods”* by potential partners' parents and family [31].

Communication difficulties, particularly surrounding sex and intimacy were another significant challenge [17, 34, 41, 43], and led to disconnection, withdrawal, and sometimes misunderstandings. Young adult cancer survivors avoiding conversations about sexual intimacy reported a decline in sexual activity [34]. This difficulty stemmed from various factors, including a lack of experience discussing sexual matters, emotional sensitivity of the topic, and the perceived stigma surrounding sexual dysfunction [34].

### 5.2 Opportunities

#### 5.2.1 Communicating for Connection

Early and upfront communication about cancer was important in developing romantic relationships [11, 31, 32, 33]. Open and honest communication about sensitive topics like family planning and fertility was reported as important by survivors, whether discussed early on or within more serious relationships. This helped to foster trust, build emotional closeness and strengthen relationships [16, 40, 41, 43]. Active listening was reported as key for couples navigating cancer treatment:

[…] You know, anyone going through cancer that’s my biggest recommendation is learn to communicate with your spouse immediately and learn to listen. I wasn’t a good listener until she was diagnosed [34].

## 6 Sexuality, Intimacy, Body Image and Fertility After Cancer

### 6.1 Challenges

#### 6.1.1 Sexual Dysfunction, Infertility, and Family Planning

Cancer and its treatment disrupted sexual wellbeing, causing a decline in desire and functioning [7, 8, 11, 16, 17, 29, 30, 31, 36, 37, 41, 45]. Physical changes resulting from cancer symptoms and treatment side effects complicated their ability to maintain sexual intimacy [8, 11, 17, 30, 34, 37, 40, 41, 44]. Males reported erectile dysfunction and lack of ejaculation. Females reported vaginal dryness, hormonal fluctuations, fatigue, body image concerns and decreased libido, making intercourse painful and unappealing [8, 11, 17, 30, 34, 37, 40, 41, 42]. For testicular cancer survivors, the experience was often characterised by a perceived threat to their masculinity, describing the removal of their testicle(s) as a *‘‘hidden secret’’* [29].

This impact was significant for AYAs who struggled to reconcile these differences with their sense of self, often associating themselves with *“older couples.”* [29, 30]. The loss of sexual confidence was compounded by concerns about their ability to meet the sexual needs of their partners:

He always brings up that we don’t want to do it. Or I don’t put effort in when we do, or I don’t initiate it’ [41].

Many worried about not finding a partner who would accept their sexual difficulties, which led to fears of romantic rejection and negatively affected self‐esteem [30, 37]:

[…] no one’s going to want someone who can’t like go down on them if it’s a woman or like give them a blowjob if it’s a guy [30].

Some experienced hurtful reactions from partners regarding their physical changes or sexual limitations:

He [partner] saw it [scar] and he was like, oh‐like I saw his face and he’s just like, oh, I didn’t know it [scar] was that big. And I just saw his reaction and [pause]‐just being in that place where I wasn’t able to do anything to tell him‐like I told him no and I wanted my shirt back [30].

AYAs faced concerns about fertility [8, 11, 16, 29, 31, 32, 37, 38, 42], with older adolescents in particular worrying about how cancer might affect their ability to have children, and the potential impact of infertility on future relationships [8]. Unpartnered survivors worried about how infertility and their changed bodies would be perceived by future partners [16]. Uncertainty about fertility was a major concern, especially for women, resulting in feelings of loss and pressure to make hasty decisions about parenthood, even if unready [38]. This uncertainty also affected survivors' feelings of desirability in romantic relationships [31, 38] and raised anxieties about adoption:

I would like to know my [fertility status], because it affects where you go with dating and future planning and things…[and the] kind of the people you consider in a relationship. […] not knowing puts me at what I feel is a disadvantage because I’m conscious of it […] [38].

Fears of passing on genetic risks weighed heavily on young survivors' minds [31, 32], leading some to avoid relationships, complicated dating or marriage prospects [31].

### 6.2 Body Image Changes and its Influence on Relationships

Cancer and its treatment considerably impacted both body image and self‐esteem, leading to challenges/barriers in forming and maintaining romantic and sexual relationships [8, 11, 16, 17, 30, 31, 32, 34, 37, 38, 41, 42]. Survivors described feeling self‐conscious about physical changes, including hair/eyebrow loss, scarring, weight fluctuations and stoma [8, 31, 37, 38], which negatively impacted their sense of self and sexuality [7, 37]:

I don’t know if it’s because without the hair and stuff, that I don’t feel attractive anymore, or my body just looks different to me [41].

While both men and women experienced body image challenges, their specific concerns aligned with traditional gender norms; women expressed anxiety about changes in their physical appearance, while men were concerned about perceived loss of masculinity or virility including uncertainty about sexual dysfunction [16, 29, 37] when navigating intimate relationships. These body related anxieties influenced how individuals approached intimate relationships:

If you’re looking to settle down, it could be a potential deal breaker to a lot of people if somehow treatment has altered your libido or altered your readiness […] [38].

### 6.3 Opportunities

#### 6.3.1 Acceptance and Future Planning

Acceptance played a central role for survivors navigating the impact of cancer on their sexuality and relationships. Some young adult survivors found that intimacy helped them reclaim a sense of normalcy and self‐acceptance after cancer:

[…] sex is a way to heal your body and your relationship to yourself. sexuality is a great way to heal your relationship with your mutilated body […] [7].

Partners who were very accepting of patient's changed appearance and sexual response helped rebuild self‐esteem and foster intimacy [11, 16, 17, 30, 32]:

He always told me I looked great, even when I thought I looked like hell [17].

This acceptance extended to the possibility of long‐term consequences from cancer, such as infertility or risk of future health issues [32].

For some, cancer prompted a re‐evaluation of family planning, with heterosexual couples placing less emphasis on biological children post‐treatment, while LGBTQ+ survivors' views remained consistent on choosing adoption or focusing on chosen family [42]. In contrast, LGBTQ+ participants generally reported more open communication and flexibility in navigating fertility and family building, often prioritising finding a partner with shared values and making joint decisions about parenthood [42].

### 6.4 Redefining Intimacy

Couples often navigated a redefined landscape of intimacy. Some couples found that facing cancer together deepened their connection, fostering a newfound appreciation of their shared journey [7, 17]. This aligned with findings from Freidus et al. [32], where partners of breast cancer survivors adapted to physical changes:

I have to be “cautious with her reconstructed breasts because she feels pain sometimes” and it took some getting used to aesthetically…it wasn’t an impediment, it was just something that took some getting used to [32].

Others redefined intimacy through connecting by kissing and hugging [11, 17, 34, 40]:

I guess we have to get a little more creative considering the main source of sexual activity [vaginal intercourse] is no longer available, which can lead to some funny conversations as well [34].

### 6.5 Sexual Empowerment

A minority of young adults described moments of empowerment in their sexuality following cancer treatment, reflected in adaptations made to intimacy that allowed them to reclaim agency over their bodies and within their relationships [30]. This included setting boundaries with partners and confronting fears about sexual limitations. Positive changes were reported such as feeling emboldened in their sexuality, which led to improved intimacy with their partner, and finding fulfilling ways to redefine intimacy [30, 34].

## 7 Partnerships and Social Support During Cancer: Balancing Caregiving, Romantic Connection and External Pressures

### 7.1 Challenges

#### 7.1.1 Shifting Sands of Support and Relational Strain

A cancer diagnosis exposed and exacerbated existing relationship issues. While some married women maintained traditional roles whilst recovering from treatment (e.g., household chores), others became dependent on their partners for physical care and emotional support ‐ sometimes straining the relationship [35]. For some, the inability to fulfill their roles as supportive partners strained relationships further [34, 37, 40].

Partners also faced the challenge in balancing their own needs with that of the survivor, at times concealing their struggles [32, 39, 40]. Partners' fear of recurrence and ongoing health concerns sometimes interfered with their ability to adequately meet the needs of the survivor, occasionally resulting in emotional withdrawal, a lack of support or feeling abandoned and resentful [11, 17, 29, 32, 35]. One survivor described it *as “a slap in the face over and over… I'm facing my own mortality and he's not here holding my hand”* [17].

For some, relationships remained strained even after treatment ended; the shift in role dynamics, coupled with changes in survivors' priorities, libido and self‐image led to frustrations, misunderstandings and disconnection [17].

#### 7.1.2 Family Dynamics and External Pressures

Families, while often a vital support system, also presented challenges for AYAs navigating romantic relationships. In a study conducted in Japan, cultural differences were found to influence these dynamics, particularly in contexts where family involvement in personal decisions is more pronounced. Traditional views on caregiving and familial responsibility such as the importance of preserving the family lineage and filial piety led some families to resist survivor's relationships due to concerns about the survivor's wellbeing or potential burden on the partner [31]:

My mother‐in‐law perceived me unfavorably because of my cancer when we married. I learned that from my partner.[31].

Partners of AYAs could also feel sidelined by well‐intentioned family members, who prioritised the patient's needs, leaving them feeling excluded [36]. Additionally, heightened social awareness experienced by adolescents created pressure to conform to societal expectations within their relationships, which potentially led to partners to *“keep up appearances”* or suppressed their own needs to maintain a certain image [36].

### 7.2 Opportunities

#### 7.2.1 Partner Support and Emotional Resilience

Partners played an essential role in supporting AYAs with cancer [8, 16, 17, 30, 35, 39, 40, 41], providing practical help such as accompanying them to appointments and assisting with daily tasks [35, 39]. Partners also offered emotional support by simply *“being there”* influencing how survivors viewed themselves to a more positive light [16, 39, 43], provided reassurance, shared quiet moments, and enabled open conversations about fears and concerns [17, 29, 39, 40, 41], which minimised conflict. This emotional support was particularly vital as AYAs navigated challenges of cancer. AYAs often confided in their partners about issues they might not have shared with family, finding in them a trusted source of advice and a sense of allegiance [39]. Furthermore, witnessing their partners navigate cancer fostered admiration and respect [17, 32, 33, 39].

## 8 Discussion

The findings from this review highlight both the challenges and opportunities that AYAs with a lived experience of cancer face in romantic relationships and sexual intimacy. The overarching theme of an ‘altered sense of self’ was identified, whereby cancer and its treatment reshaped how AYAs perceived themselves as romantic and sexual partners. While this shift in self‐perception created challenges, it also fostered opportunities for personal growth, resilience and deeper emotional connections.

The challenges identified in this review intersect with principles of attachment theory, which suggest that early relationship experiences, particularly those with primary caregivers shape how individuals approach intimacy and manage emotions in close relationships later in life [46]. Attachment patterns are typically formed during infancy or childhood, however, they can persist into adolescence and adulthood, influencing how AYAs navigate dating and intimate relationships. For AYAs navigating dating after cancer, the fear of rejection due to body changes, sexual dysfunction or infertility can create anxieties around dependence and vulnerability. This fear can lead to hesitation toward intimacy, and reluctance to pursue romantic relationships suggesting that a serious illness during adolescence and early adulthood may disrupt the development of a secure attachment style. As a result, individuals may withdraw from intimacy or struggle with dependence and autonomy in relationships [47].

Cancer‐related sexual dysfunction and infertility further complicate intimacy and relational confidence [48]. The ability to engage in sexual activity was often intertwined with feelings of desirability, masculinity/femininity, and self‐worth in relationships. These issues are similarly observed in middle‐late life stage couples, who found that cancer disrupted intimacy and forced a renegotiation of couple identity [49]. However, these challenges are amplified for AYAs who are still in the process of identity formation and relationship exploration. Older couples who likely have more established relationship patterns and already navigated key developmental milestones, typically possess a more solidified sense of self and shared life goals [50], allowing them to handle challenges such as infertility with less disruption to their relationship [51].

AYAs with cancer face unique relationship challenges, whether partnered or unpartnered. Partnered AYAs often struggle with guilt and the fear of burdening their partners, leading to communication difficulties and emotional withdrawal. While unpartnered AYAs fear judgment, challenges in when and how to disclose a cancer diagnosis in new connections, which can lead to avoidance of intimacy and isolation. For both groups, they contend with the common tension between wanting closeness while fearing vulnerability, highlighting this complexity in the context of illness [52]. Partners also play a key role in supporting AYAs with cancer which impacts relationship dynamics and psychological well‐being, yet their experiences remain relatively underexplored.

Despite these difficulties, a cancer diagnosis prompts a re‐evaluation of priorities and values, a process closely linked to identity formation and sense of self [53, 54]. Greup et al., [53] describes this as ‘post traumatic growth’, where individuals experience positive psychological changes following adversity. Confronting mortality and re‐evaluating their priorities can heighten the desire for deeper connections, and a clearer understanding of what truly matters in relationships [55]. This re‐evaluation can influence relationship dynamics, placing a higher value on emotional intimacy, authenticity, and shared values, while seeking partners who can provide a sense of understanding and support [55, 56]. Furthermore, navigating the complexities of cancer as a couple can foster open communication, strengthen problem‐solving skills, and deepen emotional intimacy, ultimately contributing to greater relationship satisfaction and resilience in managing the impact of cancer [49, 57].

### 8.1 Strengths and Limitations

Strengths of this review are that it is the first comprehensive qualitative review exploring both the challenges and opportunities AYAs face in romantic relationships and sexual intimacy. A limitation across studies is the lack of representation regarding ethnicity and sexuality. Additionally, given the sensitivity of the topics in the studies, selection bias may exist, as those more open to sharing their experiences may be more likely to participate, potentially excluding individuals struggling to adjust. Few studies disaggregated findings by age, limiting insight into how experiences may differ between young and older AYAs. Lastly, as this review was part of a broader review, supplemented with an additional search, some relevant papers may have been overlooked.

### 8.2 Implications

Existing interventions for AYAs with cancer to support romantic and sexual relationships are often part of broader support programs aimed at supporting AYAs with cancer in general. However, there are limited interventions specifically addressing concerns around romantic relationships and sexual intimacy, particularly for unpartnered AYAs. [58]. Counseling services, addressing sexual function, fertility and body image can be beneficial yet often underutilised due to stigma or lack of access [59, 60]. One targeted intervention, *Opening the Conversation* (USA) [45], was developed for young adult couples coping with reproductive and sexual health concerns after breast or gynecologic cancer. This online, couple‐based program included five weekly sessions focused on communication and coping skills, addressing body image, intimacy and family planning. Couples were invited to review and pretest intervention materials, with feedback from both heterosexual and LGBTQ+ couples highlighting the need for a flexible, accessible intervention that raises awareness of how cancer impacts relationships [45].

Furthermore, interventions that consider strategies to strengthen attachment security could be valuable for AYAs with cancer. For example, *Hand in Hand*, an attachment‐focused intervention for couples facing newly diagnosed breast cancer [61] used attachment theory based strategies to build mutual understanding of attachment behaviors, strengthen perceptions of proximity/security, and create new emotional experiences to support dyadic coping and relationship functioning. The findings found cancer related distress decreased over time in both patients and partners, though the intervention did not significantly affect distress levels post intervention or 10 months follow up. However, dyadic adjustment improved at follow up for both patients and partners suggesting that attachment focused approaches may strengthen relationship functioning during cancer even if they do not reduce individual distress. This is further supported by Heyne et al. [62] who recommend routine assessment of attachment styles (e.g., Adult Attachment Interview) and attachment informed techniques that foster secure attachment through improved emotion regulation, engaging both person with cancer and partner in the therapeutic process, and relationally focused sexual interventions that emphasise pleasure and connection rather than performance.

To provide suitable support, interventions should also consider integrating technology, social awareness, and resources from charities and healthcare organisations. For instance, dating apps, increasingly used to find romantic relationships, represent an underexplored avenue for support. Collaboration between oncology researchers and dating platforms could explore how AYAs with cancer engage with online dating and identify strategies to make these platforms more accessible and sensitive to their needs. Healthcare professionals, social workers and youth workers could also facilitate discussions on intimacy and relationships, by offering counseling for partnered and unpartnered AYAs, addressing body image, fertility, and self‐identity concerns. Additionally, workshops, online resources and peer support groups tailored to AYAs on topics like disclosure, dating, sexual intimacy, fertility preservation and family planning are also essential [8]. Charities, with established social media presence could lead campaigns to normalise conversations around relationships, sexual intimacy and body image through influencers and content creators. Peer led programs, facilitated by AYA cancer charities or youth workers in hospitals could provide support from mentors who have shared experiences, with professional input when needed.

Further research is needed to explore relationship trajectories with AYAs with poor prognosis and/or advanced illness. Additionally, exploring the impact of specific demographic factors, such as age (younger vs. older AYAs), cultural differences and LGBTQI+ perspectives remains an important area for further research.

## 9 Conclusion

Cancer and its treatment significantly impact the romantic and sexual lives of AYAs with a lived experience of cancer, for whom navigating intimacy is already a challenging developmental task. This challenge is heightened by the physical, emotional, and cultural complexities, such as body image concerns, sexual dysfunction, communication difficulties and external pressures arising from their experiences. However, these experiences also present opportunities for significant personal growth and resilience. Further research is needed to inform the development of age‐appropriate interventions and support services that might help AYAs with a lived experience of cancer to navigate sexual intimacy and relationships.

## Author Contributions

N.C.: Conceptualisation, additional searches, screening studies for inclusion in overarching review and current review, data extraction, quality assessment, writing – original draft. C.B.: Screening studies for inclusion in current review, data extraction, quality assessment, writing – review and editing. C.C.: Screening studies for inclusion in overarching review, data extraction, quality assessment, writing – review and editing. S.S.: Screening studies for inclusion in overarching review, writing – review and editing. O.H.: writing – review and editing. W.T.A.v.d.G.: writing – review and editing. M.R.‐G.: Screening studies for inclusion in overarching review, writing – review and editing. M.J.P.R.: Screening studies for inclusion in overarching review, writing – review and editing. G.E.R.: Screening studies for inclusion in overarching review, writing – review and editing. A.L.H.‐W.: Screening studies for inclusion in overarching review, writing – review and editing. K.A.: Screening studies for inclusion in overarching review, writing – review and editing. E.I.H.: Screening studies for inclusion in overarching review, writing – review and editing. L.H.L.: Screening studies for inclusion in overarching review, writing – review and editing. C.S.P.: Screening studies for inclusion in overarching review, writing – review and editing. S.H.: Screening studies for inclusion in overarching review, writing – review and editing. C.J.T.: writing – review and editing. K.J.H.: Screening studies for inclusion in overarching review, writing – review and editing. K.W.: writing – review and editing. R.W.: Screening studies for inclusion in overarching review, writing – review and editing. T.J.C.: writing – review and editing. T.L.: Screening studies for inclusion in overarching review, writing – review and editing. S.H.M.J.: Screening studies for inclusion in overarching review, writing – review and editing. A.‐S.D.: writing – review and editing.

## Ethics Statement

The authors have nothing to report.

## Conflicts of Interest

The authors declare no conflicts of interest.

## Data Availability

The authors have nothing to report.
